# Advances in Multi-Sensor Information Fusion: Theory and Applications 2017

**DOI:** 10.3390/s18041162

**Published:** 2018-04-11

**Authors:** Xue-Bo Jin, Shuli Sun, Hong Wei, Feng-Bao Yang

**Affiliations:** 1 School of Computer Information and Engineering, Beijing Technology and Business University, Beijing 100048, China; 2 School of Electronics Engineering, Heilongjiang University, Harbin 150080, China; sunsl@hlju.edu.cn; 3 Department of computer science, University of Reading, RG6 6AY Reading, UK; h.wei@reading.ac.uk; 4 School of Information and Communication Engineering, North University of China, Taiyuan 030051, China; yangfb@nuc.edu.cn

## Abstract

The information fusion technique can integrate a large amount of data and knowledge representing the same real-world object and obtain a consistent, accurate, and useful representation of that object. The data may be independent or redundant, and can be obtained by different sensors at the same time or at different times. A suitable combination of investigative methods can substantially increase the profit of information in comparison with that from a single sensor. Multi-sensor information fusion has been a key issue in sensor research since the 1970s, and it has been applied in many fields. For example, manufacturing and process control industries can generate a lot of data, which have real, actionable business value. The fusion of these data can greatly improve productivity through digitization. The goal of this special issue is to report innovative ideas and solutions for multi-sensor information fusion in the emerging applications era, focusing on development, adoption, and applications.

Information fusion technology has been in existence for several decades. At the beginning, this technology was mainly applied in military. The main reason is that at that time, the sensor was still a very expensive instrument. Military might be the only consumer who required superior performance without considering the cost.

In recent years, the application background of multi-sensor information fusion technology has undergone great changes. We have found that many civilian systems also have multi-sensor systems, such as unmanned vehicle and intelligent robot systems. Moreover, we have noticed that these systems have become a major part where the multi-sensor information fusion technology could be used, and they usually contain great research value. In these new application systems, multi-sensor information fusion technology also faces many new research issues. This is what the researchers are interested in this research field recently. We have discovered that there are many areas in which multi-sensor information fusion technology is worth investigating further. 

Our special issue was consisted of 30 papers, including the latest research results of the multi-sensor information fusion technology.

In general, these research papers were mainly divided into two parts: one is theoretical research results, and the other is the application-oriented issues. The topic discussed in theoretical research involves in-depth research on methods and theories, and it proposes new methods. The related papers mainly included three aspects: (1) a new fusion method based on the Kalman filter, including the study of various nonlinear Kalman filters, such as CKF, UKF, etc.; (2) a method based on DS evidence theory; (3) new methods for images, including video tracking, expression recognition, etc. On the other hand, we are also delighted to see that there are many papers involving solutions for various applications, which also have extremely high reading and reference value.

